# Supplementary material for: Confronting false discoveries in single-cell differential expression
Source: Nat Commun. 2021 Sep 28;12:5692. doi: 10.1038/s41467-021-25960-2 (PMC8479118; doi:10.1038/s41467-021-25960-2)
Supplement: Supplementary file 1 — Supplementary Information [file 41467_2021_25960_MOESM1_ESM.pdf]

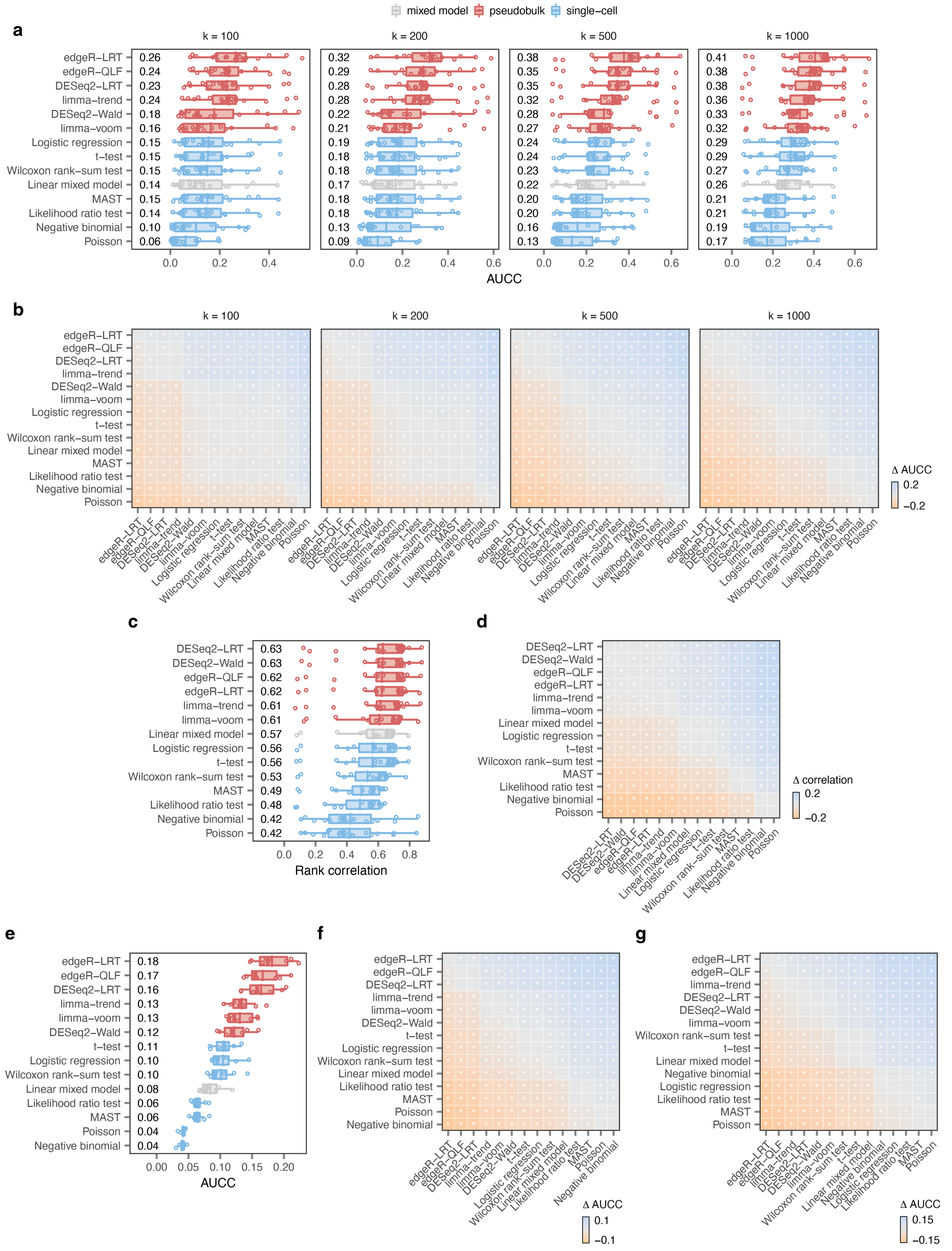
mixed model
pseudobulk
single-cell
a
k = 100
k = 200
k = 500
k = 1000
edgeR−LRT
edgeR−QLF
DESeq2−LRT
limma−trend
DESeq2−Wald
limma−voom
Logistic regression
t−test
Wilcoxon rank−sum test
Linear mixed model
MAST
Likelihood ratio test
Negative binomial
Poisson
0.26 0.24 0.23 0.24 0.18 0.16 0.15 0.15 0.15 0.14 0.15 0.14 0.10 0.06
0.32 0.29 0.28 0.28 0.22 0.21 0.19 0.18 0.18 0.17 0.18 0.18 0.13 0.09
0.38 0.35 0.35 0.32 0.28 0.27 0.24 0.24 0.23 0.22 0.20 0.20 0.16 0.13
0.41 0.38 0.38 0.36 0.33 0.32 0.29 0.29 0.27 0.26 0.21 0.21 0.19 0.17
AUCC
b
Δ AUCC
0.2
−0.2
c
DESeq2−LRT
DESeq2−Wald
edgeR−QLF
edgeR−LRT
limma−trend
limma−voom
Linear mixed model
Logistic regression
t−test
Wilcoxon rank−sum test
MAST
Likelihood ratio test
Negative binomial
Poisson
0.63 0.63 0.62 0.62 0.61 0.61 0.57 0.56 0.56 0.53 0.49 0.48 0.42 0.42
Rank correlation
d
Δ correlation
0.2
−0.2
e
edgeR−LRT
edgeR−QLF
DESeq2−LRT
limma−trend
limma−voom
DESeq2−Wald
t−test
Logistic regression
Wilcoxon rank−sum test
Linear mixed model
Likelihood ratio test
MAST
Poisson
Negative binomial
0.18 0.17 0.16 0.13 0.13 0.12 0.11 0.10 0.10 0.08 0.06 0.06 0.04 0.04
AUCC
f
Δ AUCC
0.1
−0.1
g
edgeR−LRT
edgeR−QLF
limma−trend
DESeq2−LRT
DESeq2−Wald
limma−voom
Wilcoxon rank−sum test
t−test
Linear mixed model
Negative binomial
Logistic regression
Likelihood ratio test
MAST
Poisson
Δ AUCC
0.15
−0.15

Supplementary Fig. 1 | A systematic benchmark of differential expression in single-cell transcriptomics.
a, Impact of varying the parameter k on the AUCC in the eighteen ground-truth datasets, as shown in **Fig. 1c** with $k = 500$.
b, Impact of varying the parameter k on the ΔAUCC in the eighteen ground-truth datasets, as shown in **Fig. 1d** with $k = 500$.
c, Transcriptome-wide rank correlation between single-cell and bulk RNA-seq in the eighteen ground-truth datasets shown in **a**.
d, Mean difference in the transcriptome-wide rank correlation (Δcorrelation) between the fourteen DE methods shown in **c**. Asterisks indicate comparisons with a two-tailed t-test p-value less than 0.05.
e, AUCC in eight scRNA-seq datasets with matching bulk proteomics data[1].
f, Mean ΔAUCC between the fourteen DE methods shown in **e**. Asterisks indicate comparisons with a two-tailed t-test p-value less than 0.05.
g, Mean ΔAUCC of GO term enrichment between the fourteen DE methods shown in **Fig. 1e**. Asterisks indicate comparisons with a two-tailed t-test p-value less than 0.05.

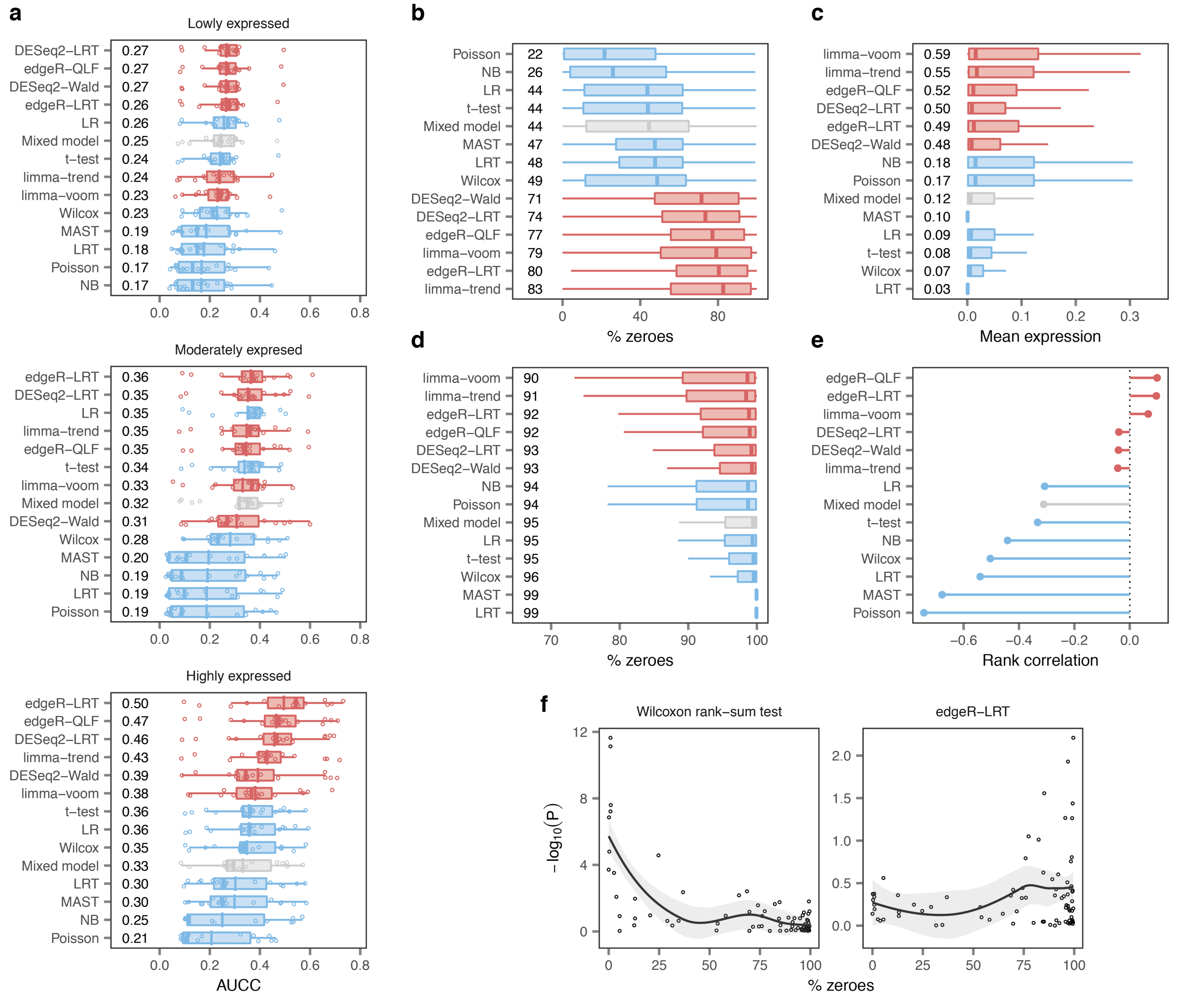

Supplementary Fig. 2 | Single-cell DE methods are biased towards highly expressed genes.

a, AUCCs across eighteen ground-truth datasets after dividing the transcriptome into terciles of lowly (top), moderately (middle), or highly (bottom) expressed genes, as shown in **Fig. 2b**.

b, Mean proportion of zero gene expression measurements for the 100 top-ranked false-positive genes from each DE method.

c, Mean expression levels of the 100 top-ranked false-negative genes from each DE method.

d, Mean proportion of zero gene expression measurements for the 100 top-ranked false-negative genes from each DE method.

e, Spearman correlation between the mean proportion of zero gene expression measurements for 80 ERCC spike-ins expressed in at least three cells and the $-\log_{10}$ p-value of differential expression assigned by each DE method.

f, Scatterplots of mean proportion of zero gene expression measurements vs. $-\log_{10}$ p-value for exemplary single-cell and pseudobulk DE methods. Trend lines and shaded areas show local polynomial regression and the 95% confidence interval, respectively.

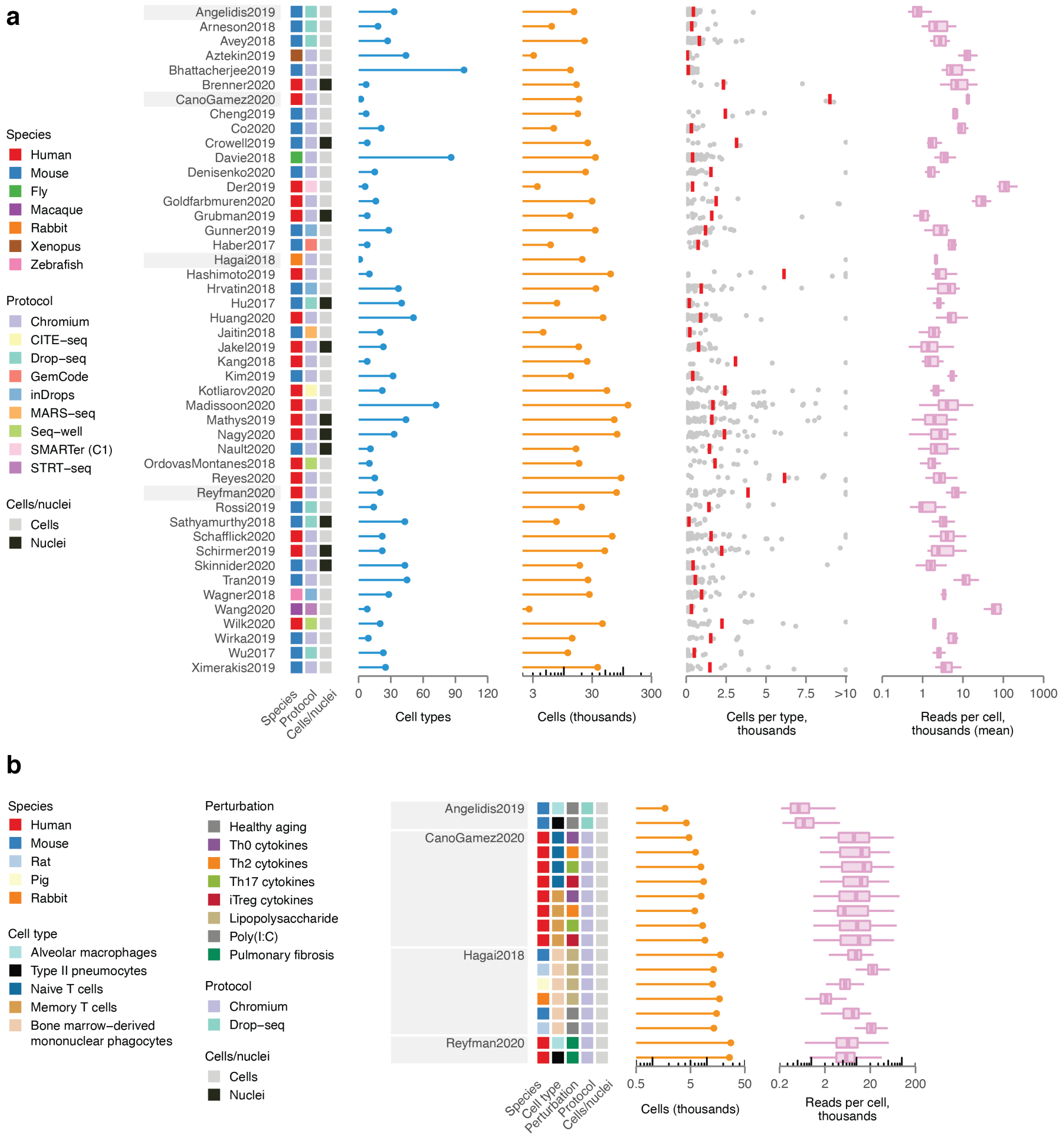

Supplementary Fig. 3 | Overview of single-cell transcriptomics datasets.

a, Overview of $n = 46$ published scRNA-seq datasets comparing two or more experimental conditions, used to systematically confirm the universality of the trends observed in analyses of individual datasets. Left, heatmap indicating the species of origin, the sequencing protocol, and whether cells or nuclei were sequenced. Right, properties of each dataset, including the total number of cell types identified in the original studies; the total number of cells sequenced; the number of cells per type (red bars indicate mean); and the mean number of reads for cells of each type. Datasets highlighted in grey contain matching bulk data and contributed to the 18 ground truth datasets shown in **b.**

b, Overview of $n = 18$ ground-truth datasets with matching scRNA-seq and bulk data, used to evaluate the biological accuracy of single-cell DE methods. Left, heatmap indicating the species of origin, the cell type under investigation and the perturbation to which it was exposed, the sequencing protocol, and whether cells or nuclei were sequenced. Right, properties of each dataset, including the total number of cells and and the number of reads per cell.

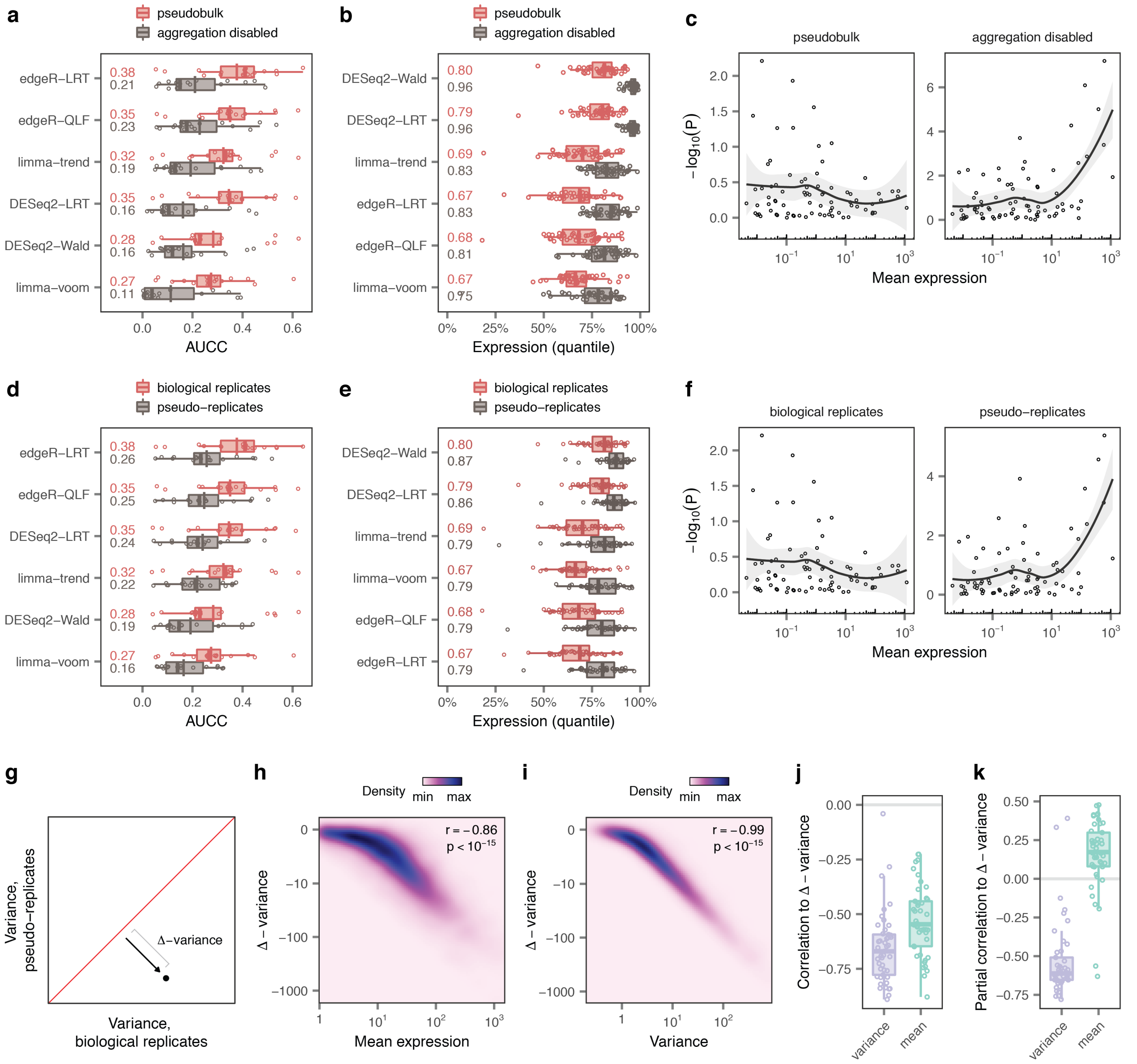
a
pseudobulk
aggregation disabled
edgeR–LRT
edgeR–QLF
limma–trend
DESeq2–LRT
DESeq2–Wald
limma–voom
AUCC
b
DESeq2–Wald
DESeq2–LRT
limma–trend
edgeR–LRT
edgeR–QLF
limma–voom
Expression (quantile)
c
−log10(P)
Mean expression
d
biological replicates
pseudo–replicates
e
f
g
Variance, pseudo–replicates
Variance, biological replicates
Δ–variance
h
Density
min
max
Δ – variance
i
Variance
j
Correlation to Δ – variance
variance
mean
k
Partial correlation to Δ – variance

Supplementary Fig. 4 | DE analysis in single-cell data must account for biological replicates.
a, AUCC of the six pseudobulk methods applied to pseudobulks or individual cells in the eighteen ground-truth datasets.
b, Mean expression levels of the 200 top-ranked genes from six pseudobulk methods applied to pseudobulks or individual cells in a collection of 46 scRNA-seq datasets.
c, Scatterplots of mean ERCC expression vs. $-\log_{10}$ p-value for an exemplary pseudobulk method, edgeR-LRT, applied to pseudobulks (left) or individual cells (right). Trend lines and shaded areas show local polynomial regression and the 95% confidence interval, respectively.
d, AUCC of the six pseudobulk methods applied to pseudobulks or pseudo-replicates in the eighteen ground-truth datasets.
e, Mean expression levels of the 200 top-ranked genes from six pseudobulk methods applied to pseudobulks or pseudo-replicates in a collection of 46 scRNA-seq datasets.
f, Scatterplots of mean ERCC expression vs. $-\log_{10}$ p-value for an exemplary pseudobulk method, edgeR-LRT, applied to pseudobulks (left) or pseudo-replicates (right). Trend lines and shaded areas show local polynomial regression and the 95% confidence interval, respectively.
g, Schematic illustrating the calculation of the Δ-variance between biological replicates and pseudo-replicates.
h, Correlation between mean expression and Δ-variance for 10,448 genes with mean expression $\geq$ 1 CPM in the dataset of mouse bone marrow mononuclear cells stimulated with poly-I:C. Mean expression is strongly correlated with Δ-variance, such that the variance of highly expressed genes is disproportionately underestimated when ignoring information about biological replicates.
i, Correlation between expression variance and Δ-variance for 10,448 genes with mean expression $\geq$ 1 CPM in the dataset of mouse bone marrow mononuclear cells stimulated with poly-I:C. Variance is even more strongly correlated with Δ-variance than mean expression, such that the most variable genes are disproportionately underestimated when ignoring information about biological replicate.
j, Correlation between mean expression levels or expression variance and Δ-variance in 46 scRNA-seq datasets. Variance is even more strongly correlated with Δ-variance than mean expression across a large compendium of datasets, corroborating the trends shown in **h-i**.
k, Partial correlation between mean expression and Δ-variance, controlling for variance, or between variance and Δ-variance, controlling for mean expression. The variance of gene expression is the primary determinant of Δ-variance, implying that failing to account for biological replicates introduces a bias towards highly expressed genes because these genes are also more variable.

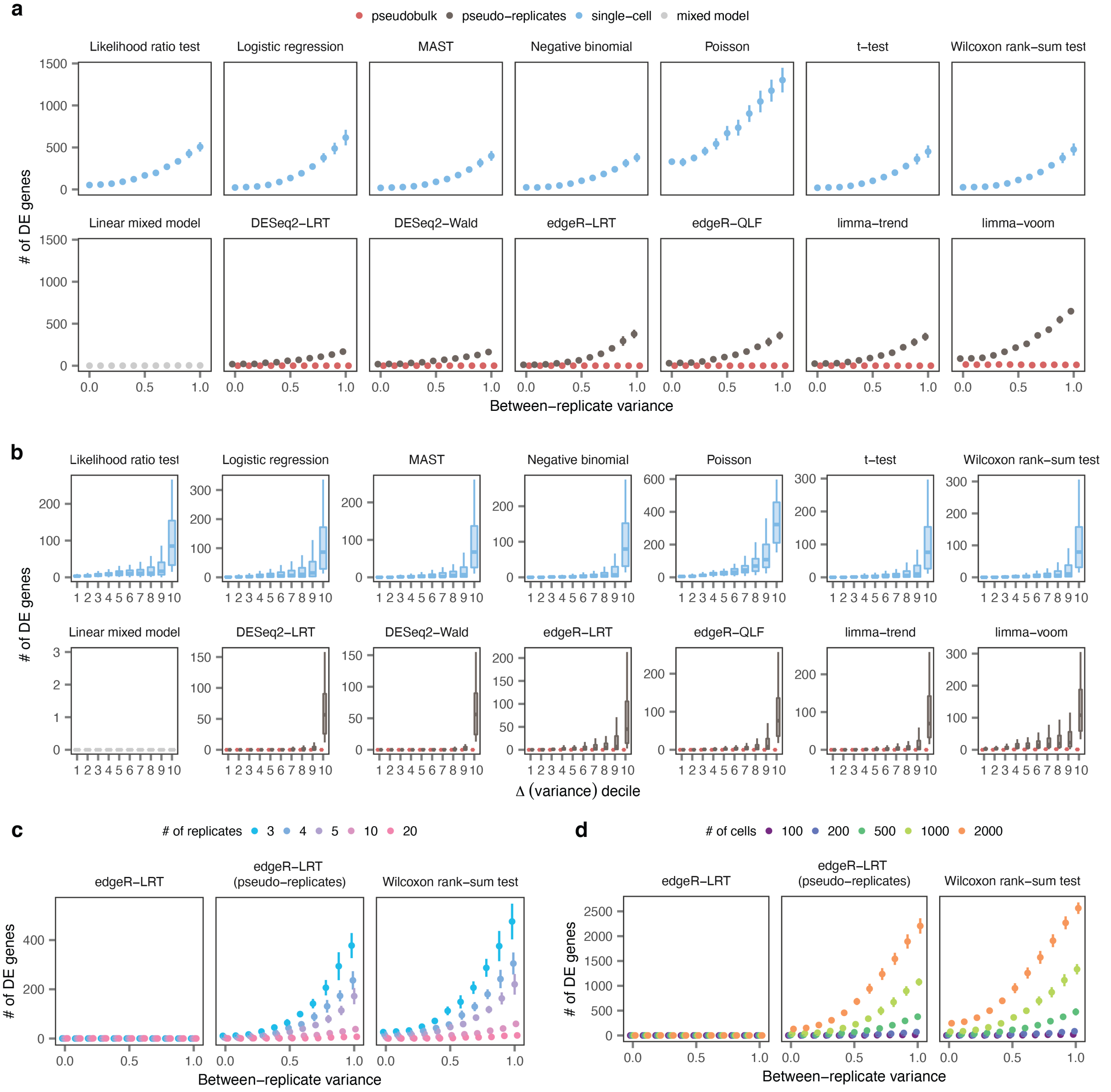

Supplementary Fig. 5 | Simulation studies expose false discoveries in single-cell DE.

a, Number of DE genes detected in stimulation experiments with varying degrees of heterogeneity between replicates by all DE methods. Points and error bars show the mean and standard deviation of ten independent simulations.

b, Number of DE genes detected by the tests shown in a for genes divided into deciles by the magnitude of the change in variance between biological replicates and pseudo-replicates (Δ-variance).

c, Number of DE genes detected by a representative single-cell DE method, a representative pseudobulk method, and the same pseudobulk method applied to pseudo-replicates, when varying the total number of replicates in the simulated dataset. Points and error bars show the mean and standard deviation of ten independent simulations.

d, Number of DE genes detected by a representative single-cell DE method, a representative pseudobulk method, and the same pseudobulk method applied to pseudo-replicates, when varying the total number of cells in the simulated dataset. Points and error bars show the mean and standard deviation of ten independent simulations.

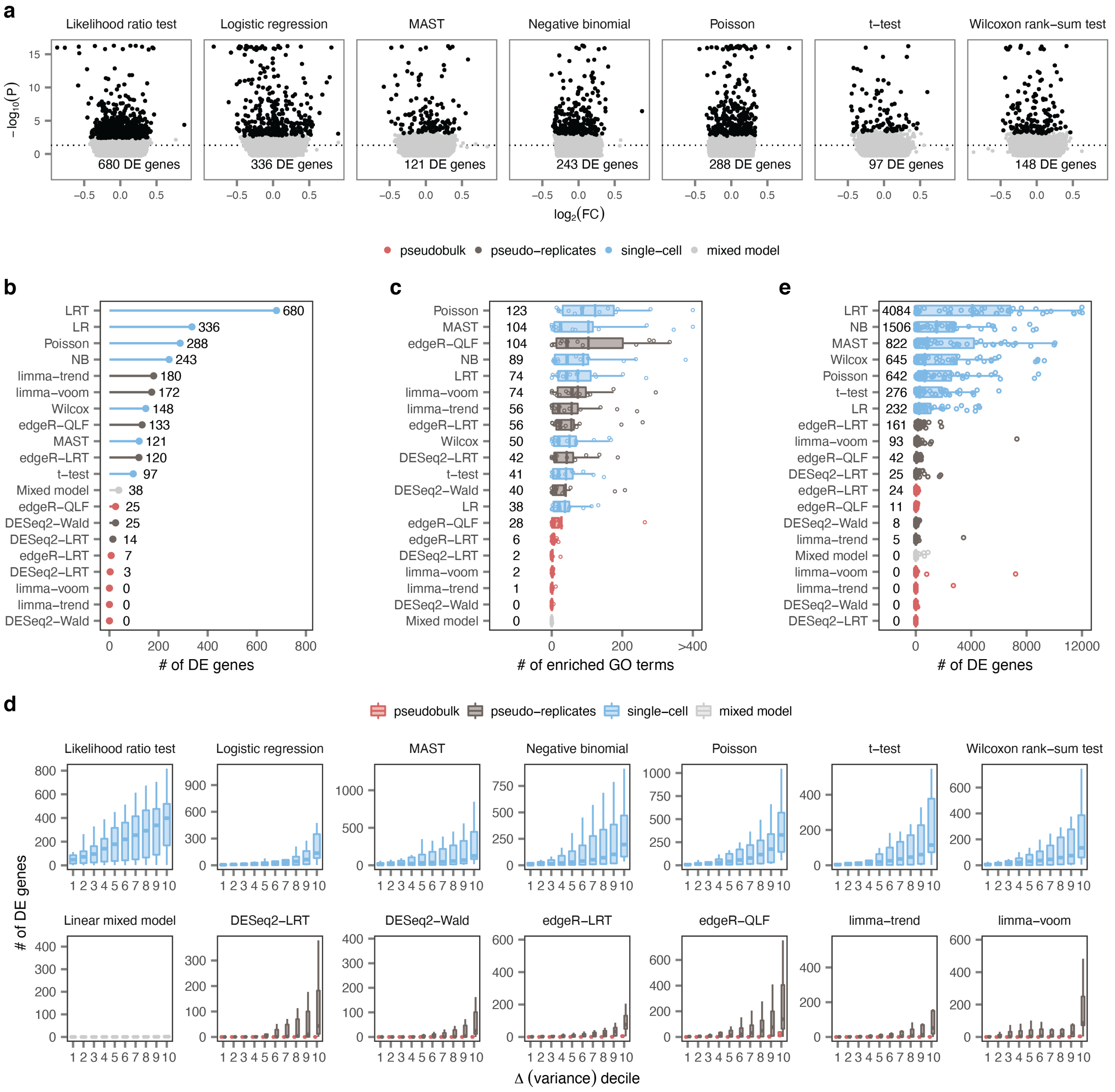

Supplementary Fig. 6 | False discoveries in single-cell and spatial transcriptomics data.

a, Volcano plots showing DE between T cells from random groups of unstimulated controls drawn from Kang et al.[2] using seven single-cell DE methods.

b, Number of DE genes detected by all DE methods in unstimulated T cells.

c, Number of GO terms enriched at 5% FDR among DE genes identified in comparisons of random groups of unstimulated controls from fourteen scRNA-seq studies with at least six control samples.

d, Number of DE genes in comparisons of random groups of unstimulated controls from fourteen scRNA-seq studies with at least six control samples, as shown in **Fig. 4e**, for genes divided into deciles by the magnitude of the change in variance between biological replicates and pseudo-replicates (Δ-variance).

e, Number of DE genes detected by all DE methods within spinal cord regions from control mice profiled by spatial transcriptomics[3].

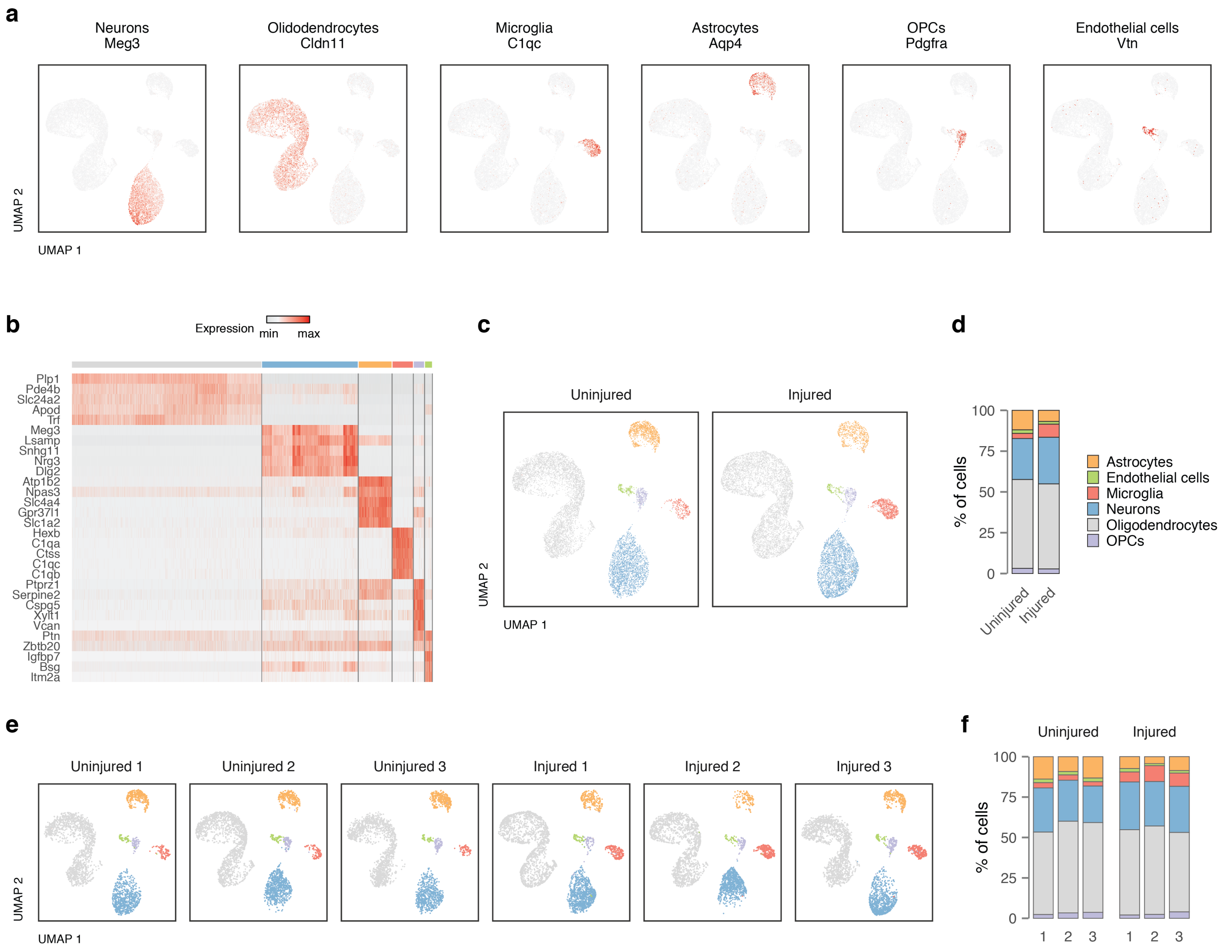

Supplementary Fig. 7 | Single-nucleus RNA-seq of the injured mouse lumbar spinal cord.
a, Expression of key marker genes for the six major cell types of the lumbar spinal cord across 19,237 individual nuclei.
b, Top five marker genes for each major cell type of the lumbar spinal cord.
c, UMAP visualization of 19,237 nuclei, showing detection of the major cell types of the lumbar spinal cord across experimental conditions.
d, Proportion of cells of each major cell type detected in either experimental condition.
e, UMAP visualization of 19,237 nuclei, showing detection of the major cell types of the lumbar spinal cord across individual replicates.
f, Proportion of cells of each major cell type detected in each individual replicate.

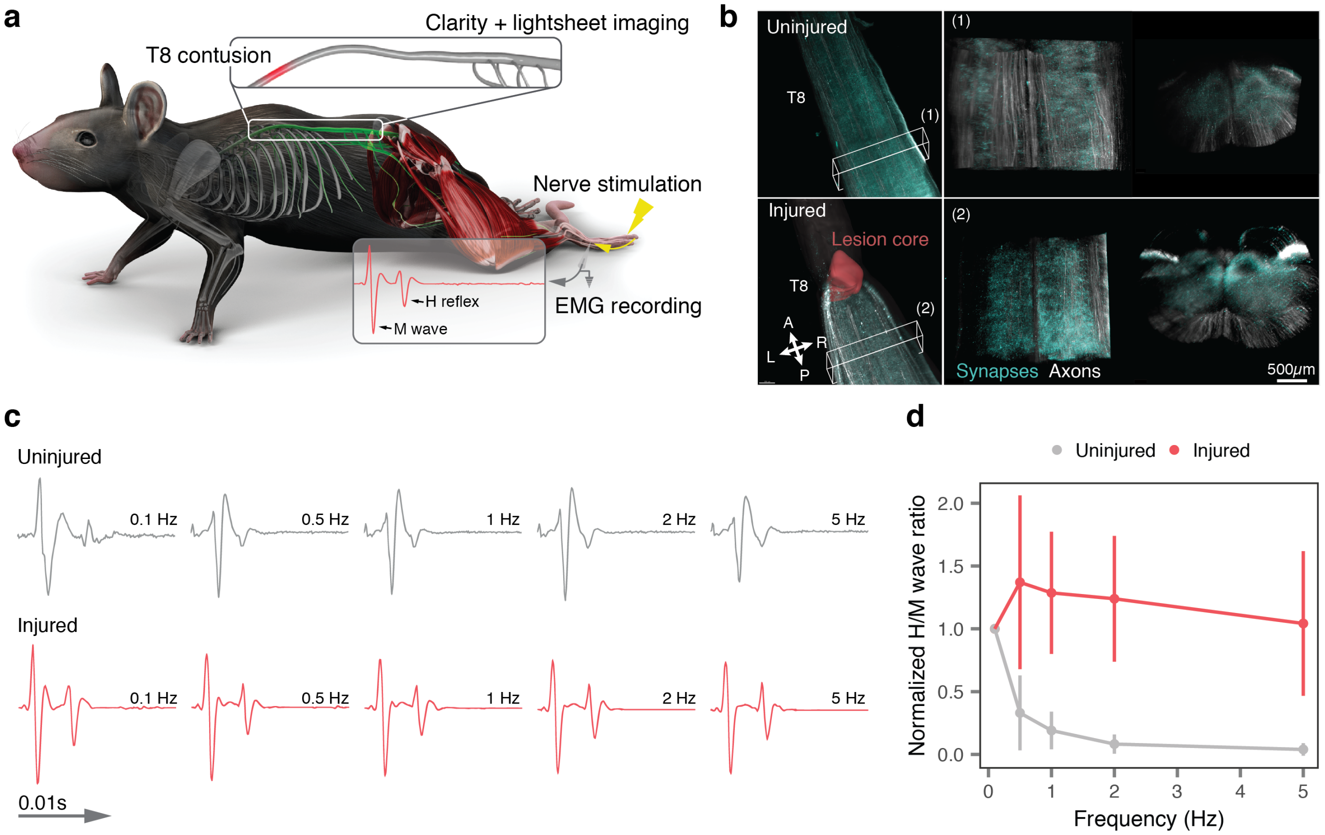

Supplementary Fig. 8 | Spasticity and hyperexcitability in the injured mouse lumbar spinal cord.
a, Schematic illustration of electrophysiology experimental design. Inset demonstrates persistent amplitude at increasing frequency of the H-reflex in response to plantar stimulation, reflecting hyperexcitability.
b, Synapses and projections of *Vglut2*^{ON} neurons in the mouse lumbar spinal cord before and after SCI.
c, Individual traces from representative injured and uninjured mice at each tested frequency, including 0.1, 0.5, 1, 2, and 5 Hz. We observed a persistent H-reflex in only injured animals, indicating hyperexcitability.
d, Quantification of electrophysiology traces as shown in **c,** demonstrating persistence of the H-reflex in the injured group. Points and error bars show mean and standard deviation, respectively, for n = 6 biologically independent animals examined with 12 repeated measures for each condition.
Source data are provided as a Source Data file.

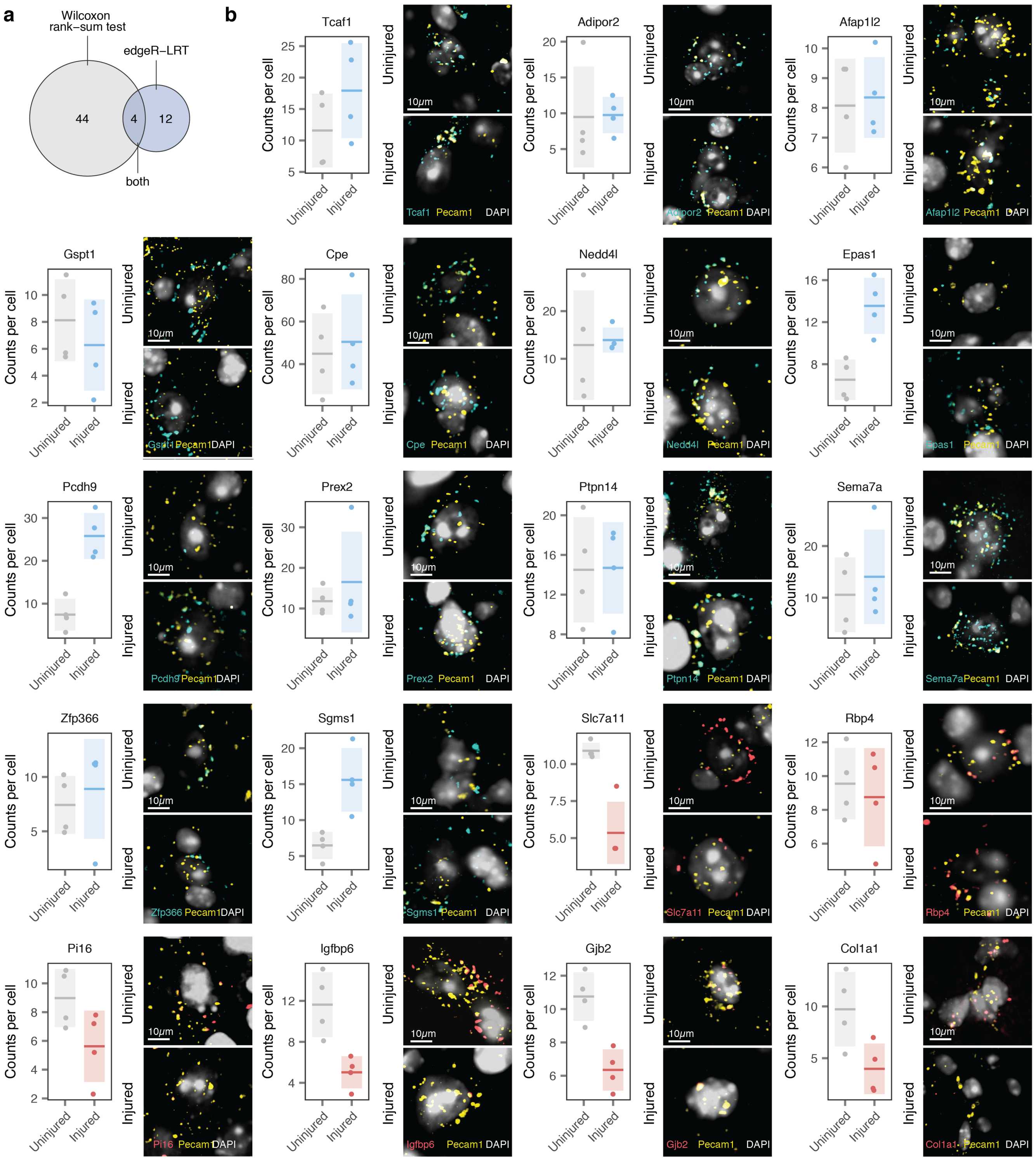

Supplementary Fig. 9 | *In vivo* validation of single-cell DE analysis by RNAscope.

a, Overlap between genes identified as DE within endothelial cells of the injured mouse lumbar spinal cord by edgeR-LRT and the Wilcoxon rank-sum test.

b, RNAscope quantification, left, and representative images, right, for nineteen genes identified as DE exclusively by edgeR-LRT or the Wilcoxon rank-sum test. Horizontal line and shaded area show the mean and standard deviation, respectively.

Source data are provided as a Source Data file.

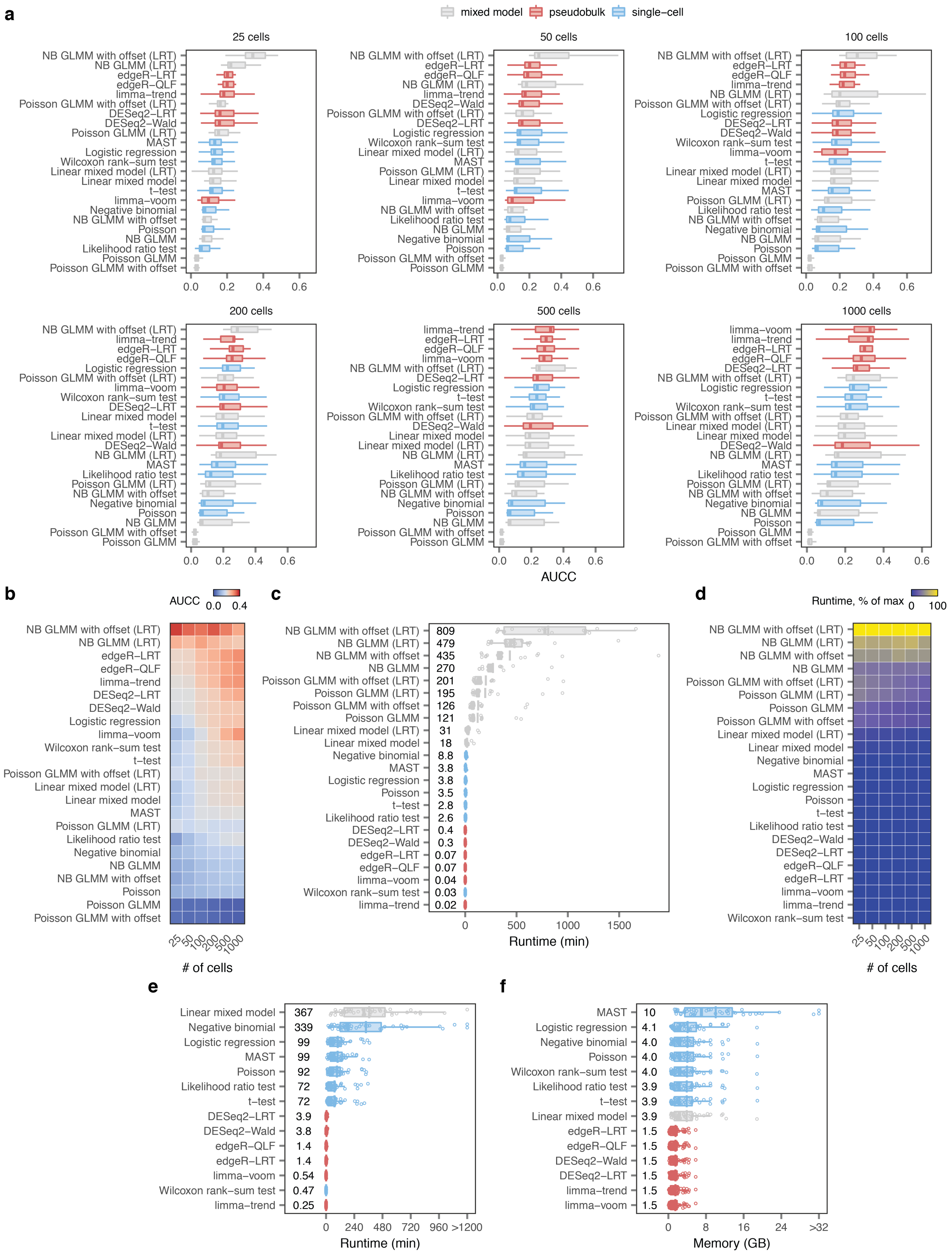
a
mixed model
pseudobulk
single−cell
25 cells
50 cells
100 cells
200 cells
500 cells
1000 cells
AUCC
b
AUCC
0.0
0.4
# of cells
c
NB GLMM with offset (LRT) 809
NB GLMM (LRT) 479
NB GLMM with offset 435
NB GLMM 270
Poisson GLMM with offset (LRT) 201
Poisson GLMM (LRT) 195
Poisson GLMM with offset 126
Poisson GLMM 121
Linear mixed model (LRT) 31
Linear mixed model 18
Negative binomial 8.8
MAST 3.8
Logistic regression 3.8
Poisson 3.5
t−test 2.8
Likelihood ratio test 2.6
DESeq2−LRT 0.4
DESeq2−Wald 0.3
edgeR−LRT 0.07
edgeR−QLF 0.07
limma−voom 0.04
Wilcoxon rank−sum test 0.03
limma−trend 0.02
Runtime (min)
d
Runtime, % of max
# of cells
e
Linear mixed model 367
Negative binomial 339
Logistic regression 99
MAST 99
Poisson 92
Likelihood ratio test 72
t−test 72
DESeq2−LRT 3.9
DESeq2−Wald 3.8
edgeR−QLF 1.4
edgeR−LRT 1.4
limma−voom 0.54
Wilcoxon rank−sum test 0.47
limma−trend 0.25
Runtime (min)
f
MAST 10
Logistic regression 4.1
Negative binomial 4.0
Poisson 4.0
Wilcoxon rank−sum test 4.0
Likelihood ratio test 3.9
t−test 3.9
Linear mixed model 3.9
edgeR−LRT 1.5
edgeR−QLF 1.5
DESeq2−Wald 1.5
DESeq2−LRT 1.5
limma−trend 1.5
limma−voom 1.5
Memory (GB)

Supplementary Fig. 10 | Single-cell DE analysis with generalized linear mixed models.
a, AUCC for ten different generalized linear mixed models (GLMMs), varying in the choice of link function (identity, Poisson, or negative binomial, NB); method used to evaluate statistical significance (Wald test or likelihood ratio test, LRT), and presence of an offset term, in samples of between 25 and 1,000 cells from the eighteen ground-truth datasets shown in **Fig. 1c**, and compared to the fourteen DE methods shown in the same panel.
b, As in **a,** but showing the mean AUCC as a function of the number of cells sampled for each DE method.
c, Runtime in minutes for the ten GLMMs shown in a in samples of 1,000 cells. The top-performing GLMM required a mean of 13.5 h per cell type to perform DE analysis.
d, Runtime of the ten GLMMs and the fourteen DE methods shown in **Fig. 1c**, shown as a percentage of the maximum runtime, as a function of the number of cells sampled.
e, Runtime in minutes of the fourteen DE methods shown in **Fig. 1c** across 46 scRNA-seq datasets.
f, Maximum memory required in gigabytes by the fourteen DE methods shown in **Fig. 1c** across 46 scRNA-seq datasets.

References

1. Cano-Gamez, E. *et al.* Single-cell transcriptomics identifies an effectorness gradient shaping the response of CD4+ T cells to cytokines. *Nat. Commun.* **11,** 1801 (2020).
2. Kang, H. M. *et al.* Multiplexed droplet single-cell RNA-sequencing using natural genetic variation. *Nat. Biotechnol.* **36,** 89–94 (2018).
3. Maniatis, S. *et al.* Spatiotemporal dynamics of molecular pathology in amyotrophic lateral sclerosis. *Science* **364,** 89–93 (2019).
