# Supplementary material for: Confronting false discoveries in single-cell differential expression
Source: Nat Commun. 2021 Sep 28;12:5692. doi: 10.1038/s41467-021-25960-2 (PMC8479118; doi:10.1038/s41467-021-25960-2)
Supplement: Supplementary file 2 — Description of Additional Supplementary Files [file 41467_2021_25960_MOESM2_ESM.docx]

Description of Additional Supplementary Files

Title: Supplementary Software.

Description: Libra R package.
