# Supplementary material for: Confronting false discoveries in single-cell differential expression
Source: Nat Commun. 2021 Sep 28;12:5692. doi: 10.1038/s41467-021-25960-2 (PMC8479118; doi:10.1038/s41467-021-25960-2)
Supplement: Supplementary file 4 — Reporting Summary [file 41467_2021_25960_MOESM4_ESM.pdf]

nature research

Corresponding author(s): Gregoire Courtine, Michael Skinnider

Last updated by author(s): 08-04-2021

Reporting Summary

Nature Research wishes to improve the reproducibility of the work that we publish. This form provides structure for consistency and transparency in reporting. For further information on Nature Research policies, see our Editorial Policies and the Editorial Policy Checklist.

Statistics

For all statistical analyses, confirm that the following items are present in the figure legend, table legend, main text, or Methods section.

n/a	Confirmed	
☐	☒	The exact sample size (n) for each experimental group/condition, given as a discrete number and unit of measurement
☐	☒	A statement on whether measurements were taken from distinct samples or whether the same sample was measured repeatedly
☐	☒	The statistical test(s) used AND whether they are one- or two-sided<br>*Only common tests should be described solely by name; describe more complex techniques in the Methods section.*
☐	☒	A description of all covariates tested
☐	☒	A description of any assumptions or corrections, such as tests of normality and adjustment for multiple comparisons
☐	☒	A full description of the statistical parameters including central tendency (e.g. means) or other basic estimates (e.g. regression coefficient) AND variation (e.g. standard deviation) or associated estimates of uncertainty (e.g. confidence intervals)
☐	☒	For null hypothesis testing, the test statistic (e.g. F, t, r) with confidence intervals, effect sizes, degrees of freedom and P value noted<br>*Give P values as exact values whenever suitable.*
☒	☐	For Bayesian analysis, information on the choice of priors and Markov chain Monte Carlo settings
☒	☐	For hierarchical and complex designs, identification of the appropriate level for tests and full reporting of outcomes
☐	☒	Estimates of effect sizes (e.g. Cohen's d, Pearson's r), indicating how they were calculated

Our web collection on statistics for biologists contains articles on many of the points above.

Software and code

Policy information about availability of computer code

Data collection	Data were collected using Zen2 Black (Zeiss), Imaris (9.1.2, 64Bit, Bitplane).
Data analysis	We provide an R package, Libra, implementing all methods for DE analysis discussed in this study within a consistent interface. The Libra package is available from GitHub (https://github.com/neurorestore/Libra) and as Supplementary Software 1. In addition, the R source code used to perform data analysis is available from GitHub at https://github.com/neurorestore/DE-analysis.

For manuscripts utilizing custom algorithms or software that are central to the research but not yet described in published literature, software must be made available to editors and reviewers. We strongly encourage code deposition in a community repository (e.g. GitHub). See the Nature Research guidelines for submitting code & software for further information.

Data

Policy information about availability of data

All manuscripts must include a data availability statement. This statement should provide the following information, where applicable:

- Accession codes, unique identifiers, or web links for publicly available datasets
- A list of figures that have associated raw data
- A description of any restrictions on data availability

Raw sequencing data and count matrices have been deposited to the Gene Expression Omnibus under accession code GSE165003 (https://www.ncbi.nlm.nih.gov/geo/query/acc.cgi?acc=GSE165003). The 18 'ground truth' datasets are available from Zenodo at http://dx.doi.org/10.5281/zenodo.5048449.

Field-specific reporting

Please select the one below that is the best fit for your research. If you are not sure, read the appropriate sections before making your selection.

- [x] Life sciences
- [ ] Behavioural & social sciences
- [ ] Ecological, evolutionary & environmental sciences

For a reference copy of the document with all sections, see nature.com/documents/nr-reporting-summary-flat.pdf

Life sciences study design

All studies must disclose on these points even when the disclosure is negative.

Sample size	We assembled a compendium of 46 published single-cell or single-nucleus RNA-seq studies (Supplementary Fig. 3), and performed DE analyses across this compendium to establish the generality of our conclusions. The sample sizes for each of these studies were established by the authors of the original studies.
Data exclusions	For publications containing more than one comparison, only a single comparison was retained, as described in detail in the Methods section. We retained the comparison involving the greatest number of cells, and used the most fine-grained cell type annotations provided by the authors of the original studies. When count matrices did not use gene symbols, the provided identifiers were mapped to gene symbols, and counts summed across genes mapping to identical symbols. Only cell types with at least three cells in each condition were subjected to DE analysis, and genes detected in less than three cells were removed.
Replication	Our findings were replicated in 46 published single-cell transcriptomics datasets.
Randomization	Randomization was not relevant as the study involved re-analysis of published datasets.
Blinding	Blinding was not relevant as the study involved re-analysis of published datasets.

Reporting for specific materials, systems and methods

We require information from authors about some types of materials, experimental systems and methods used in many studies. Here, indicate whether each material, system or method listed is relevant to your study. If you are not sure if a list item applies to your research, read the appropriate section before selecting a response.

Materials & experimental systems

n/a	Involved in the study	
☐	☒	Antibodies
☒	☐	Eukaryotic cell lines
☒	☐	Palaeontology and archaeology
☐	☒	Animals and other organisms
☒	☐	Human research participants
☒	☐	Clinical data
☒	☐	Dual use research of concern

Methods

n/a	Involved in the study	
☒	☐	ChIP-seq
☒	☐	Flow cytometry
☒	☐	MRI-based neuroimaging

Antibodies

Antibodies used	Primary antibodies were: rat anti-Pecam1 (BD Biosciences 550274, 1:200). Secondary antibodies were: Alexa Fluor 555 Goat Anti Rat (1:200, Life Technologies, A21434).
Validation	We used commercial antibodies. All antibodies have been guaranteed and validated by the manufacturers. We also included positive and negative controls for every histological procedure. The concentration of each antibody was tested before use and confirmed based on the morphology of positive signal.

Animals and other organisms

Policy information about studies involving animals; ARRIVE guidelines recommended for reporting animal research

Laboratory animals	Experiments were conducted on adult male or female C57BL/6 mice (15-35 g body weight, 12-30 weeks of age). Vglut2:Cre (Jackson Laboratory 016963) transgenic mice were used and maintained on a mixed genetic background (129/C57BL/6).
Wild animals	No wild animals were used in the study.
Field-collected samples	No field collected samples were used in the study.

Ethics oversight	All procedures and surgeries were approved by the Veterinary Office of the Canton of Geneva (Switzerland).

Note that full information on the approval of the study protocol must also be provided in the manuscript.
